# Unique B-1 cells specific for both *N*-pyrrolated proteins and DNA evolve with apolipoprotein E deficiency

**DOI:** 10.1016/j.jbc.2022.101582

**Published:** 2022-01-11

**Authors:** Sei-Young Lim, Kosuke Yamaguchi, Masanori Itakura, Miho Chikazawa, Tomonari Matsuda, Koji Uchida

**Affiliations:** 1 Graduate School of Agricultural and Life Sciences, The University of Tokyo, Tokyo, Japan; 2 Research Center for Environmental Quality Management, Kyoto University, Otsu, Shiga, Japan; 3 Japan Agency for Medical Research and Development, CREST, Tokyo, Japan

**Keywords:** posttranslational modification (PTM), apolipoprotein E deficiency, *N*^*ε*^-pyrrole-L-lysine, innate immunity, autoimmunity, anti-DNA antibody, immunoglobulin M (IgM), B cell receptor (BCR), lipid peroxidation, monoclonal antibody, Abs, antibodies, Ag, antigen, apoE, apolipoprotein E, apoE^−/−^, apoE-deficient, BCR, B cell receptor, BSA, bovine serum albumin, FR1∼4, framework region 1∼4, HCDR1∼3, heavy chain complementarity-determining region1∼3, IGHD, immunoglobulin heavy chain diversity, IGHJ, immunoglobulin heavy chain joining, IGHV, immunoglobulin heavy chain variable, IGKJ, immunoglobulin κ chain joining, IGKV, immunoglobulin κ chain variable, IgM, immunoglobulin M, LCDR1∼3, light chain complementarity-determining region 1∼3, LPS, lipopolysaccharide, mAbs, monoclonal antibodies, N-addition, non-templated-nucleotide addition, N nucleotide, non-templated nucleotide, oxLDL, oxidized LDL, PerC, peritoneal cavity, Pyr^+^, pyrBSA-binding, pyrBSA, *N*-pyrrolated BSA, pyrP, pyrrolated proteins, pyrK, *N*^*ε*^-pyrrole-L-lysine, SPL, spleen, VH, heavy chain variable region, VL, light chain variable region, WT, wild-type

## Abstract

Lysine *N*-pyrrolation, a posttranslational modification, which converts lysine residues to *N*^*ε*^-pyrrole-L-lysine, imparts electronegative properties to proteins, causing them to mimic DNA. Apolipoprotein E (apoE) has been identified as a soluble receptor for pyrrolated proteins (pyrP), and accelerated lysine *N*-pyrrolation has been observed in apoE-deficient (apoE^−/−^) hyperlipidemic mice. However, the impact of pyrP accumulation consequent to apoE deficiency on the innate immune response remains unclear. Here, we investigated B-1a cells known to produce germline-encoded immunoglobulin M (IgM) from mice deficient in apoE and identified a particular cell population that specifically produces IgM antibodies against pyrP and DNA. We demonstrated an expansion of B-1a cells involved in IgM production in the peritoneal cavity of apoE^−/−^ mice compared with wild-type mice, consistent with a progressive increase of IgM response in the mouse sera. We found that pyrP exhibited preferential binding to B-1a cells and facilitated the production of IgM. B cell receptor analysis of pyrP-specific B-1a cells showed restricted usage of gene segments selected from the germline gene set; most sequences contained high levels of non-templated-nucleotide additions (N-additions) that could contribute to junctional diversity of B cell receptors. Finally, we report that a subset of monoclonal IgM antibodies against pyrP/DNA established from the apoE^−/−^ mice also contained abundant N-additions. These results suggest that the accumulation of pyrP due to apoE deficiency may influence clonal diversity in the pyrP-specific B cell repertoire. The discovery of these unique B-1a cells for pyrP/DNA provides a key link connecting covalent protein modification, lipoprotein metabolism, and innate immunity.

The covalent modification of proteins has been implicated in a variety of diseases, including atherosclerosis (1, 2). Important molecules that cause covalent modification of proteins include oxidized fatty acids and glycolysis intermediates, many of which are aldehydic species that can mediate cell damage and disrupt important cell functions (3, 4). The *ε*-amino group of lysine is one of the major targets of covalent protein modification with aldehydes, producing aldehyde-amino acid adducts with heterocyclic structural motifs. The formation of these adducts has a profound effect on the chemical properties of proteins with important functional and regulatory consequences.

Natural antibodies (NAbs), primarily immunoglobulin M (IgM), protect against exogenous and endogenous antigens in order to maintain homeostasis (5, 6). They are primarily produced by B-1 cells, a subtype of B cell lymphocytes involved in the humoral immune response, even in the absence of antigen stimulation, allowing the B-1 cells to provide existing immediate protection against antigens (7, 8, 9). They are defined as germline-encoded immunoglobulins and are characterized by a multispecificity to structures, such as nucleic acid, carbohydrates, and phospholipids (10, 11, 12). Previous studies have shown that covalent protein modification results in NAbs-reactive self-antigens, such as oxidized low-density lipoproteins and advanced glycation end products (6, 11). Our recent studies have also shown that proteins modified with a toxic aldehyde, acrolein, could be targets for NAbs (13). The presence of these NAbs may reflect the ubiquitous formation of covalently modified proteins as a result of various intracellular and extracellular events.

Lysine *N*-pyrrolation is a unique covalent modification that converts lysine residues to *N*^*ε*^-pyrrole-L-lysine (pyrK) (Fig. 1*A*) (14). Since *N*-pyrrolation is a charge-neutralizing reaction, the lysine *N*-pyrrolation of proteins is accompanied by an increase in the net negative charges of the protein. In addition, proteins acquire an electrical conductivity *via* lysine *N*-pyrrolation (14). Interestingly, pyrrolated proteins (pyrP) are recognized by anti-DNA Abs, and the underlying mechanism of the affinity for anti-DNA Abs is presumed to be due to changes in the electrical properties of the proteins *via* lysine *N*-pyrrolation (14). It has also been shown that pyrP are stained by DNA intercalators, such as SYBR Green I, due probably to stacking interactions between the pyrrole rings and/or between the pyrrole rings and aromatic amino acid residues (14). These data indicated that the lysine *N*-pyrrolation is a physiologically relevant posttranslational modification that gives rise to DNA mimic proteins. They also presented a fascinating hypothesis that the accumulation of pyrP might be directly linked to the production of autoantibodies (autoAbs) for DNA. In connection with this hypothesis, we recently identified apolipoprotein E (apoE) as a soluble receptor for pyrP and observed accelerated lysine *N*-pyrrolation associated with the accumulation of antibodies (Abs) against pyrP and DNA in apoE-deficient (apoE^−/−^) hyperlipidemia mice (Fig. 1*B*) (15). However, the mechanism of the immune responses to pyrP associated with the apoE deficiency remains unclear.

**Figure 1:**
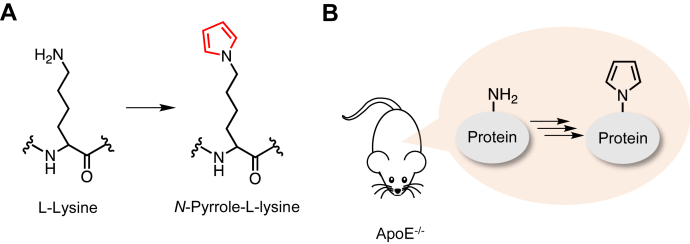
**Schematic representation of formation and accumulation of pyrP in apoE**^**−/−**^**mice.***A*, the scheme of lysine *N*-pyrrolation (*B*) Accumulation of pyrP in apoE^−/−^ mice.

In the current study, to examine the impact of the apoE deficiency on the innate immune response, we investigated pyrrolation-specific B cells that produce IgM in response to pyrP in wild-type (WT) and apoE^−/−^ mice and identified unique B-1a cells. Based on this finding, we analyzed the B cell receptor (BCR) repertoire of pyrrolation-responsive B cells and characterized the heavy chain variable region (VH), focusing on the preferential V, D, and J gene usage and non-templated-nucleotide insertion (N-addition). In addition, we established a subset of hybridoma clones and characterized the specificity and structural properties of IgM Abs in detail. Evidence of the presence of bispecific IgM-BCRs highlights pyrP in driving the expansion of B-1a cells and production of bispecific IgM Abs for pyrP/DNA.

## Results

### Innate immune response to pyrP

**Figure 2:**
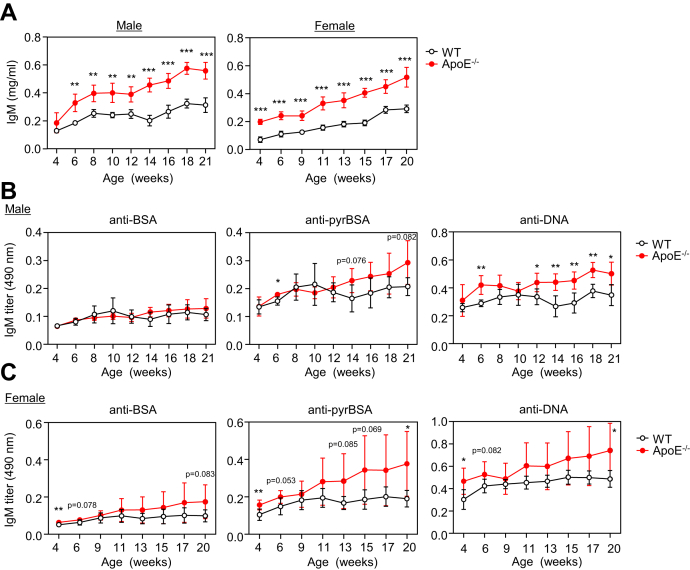
**Innate immune response to pyrP in WT and apoE**^**−/−**^**mice**. *A*, age-dependent elevation of IgM production in WT and apoE^−/−^ mice. Total IgM levels in the sera of up to 21-week-old male mice (*left panel*) and up to 20-week-old female mice (*right panel*) were measured by sandwich ELISA. The data are shown as the mean ± SD (n = 4∼6). Differences were analyzed by the unpaired Student’s *t* test; ∗∗*p* < 0.01; ∗∗∗*p* < 0.001 for each time point. *B* and *C*, age-dependent elevation of the IgM titers in the sera of male (*B*) and female mice (*C*) against BSA (*left panels*), pyrBSA (*middle panels*), and DNA (*right panels*). The IgM titers were determined by direct antigen ELISA with the sera of the WT and apoE^−/−^ mice. BSA, pyrBSA, and DNA were used as the coating antigens. The data are shown as the mean ± SD (n = 4∼6). Differences were analyzed by the unpaired Student’s *t* test; ∗*p* < 0.05; ∗∗*p* < 0.01 for each time point.

Based on our previous finding that the apoE deficiency leads to a significant accumulation of pyrP in mice (15), we first examined changes in the serum IgM levels in the WT and apoE^−/−^ mice. Although age-dependent increases in the IgM levels were detected in both the WT and apoE^−/−^ mice, significantly higher levels of serum IgM were observed in the apoE^−/−^ mice than in the WT mice throughout the age range (Fig. 2*A*). When screened for reactive aldehydes as a source of the innate epitopes, apoE^−/−^ mice showed significantly higher IgM titers against modified proteins, such as pyrrolated BSA (pyrBSA), compared with the WT mice (Fig. S1). When the anti-pyrBSA IgM titers in the sera of the WT and apoE^−/−^ mice were measured, an increasing tendency was observed in the apoE^−/−^ mice as compared with the WT mice (Fig. 2, *B* and *C*). The levels of the anti-DNA IgM titers were also higher in the apoE^−/−^ mice than those in the control mice. Of interest, both the WT and apoE^−/−^ female mice showed slightly higher anti-pyrBSA IgM titers than the males.

### Changes in B cell subsets and their response to pyrP

**Figure 3:**
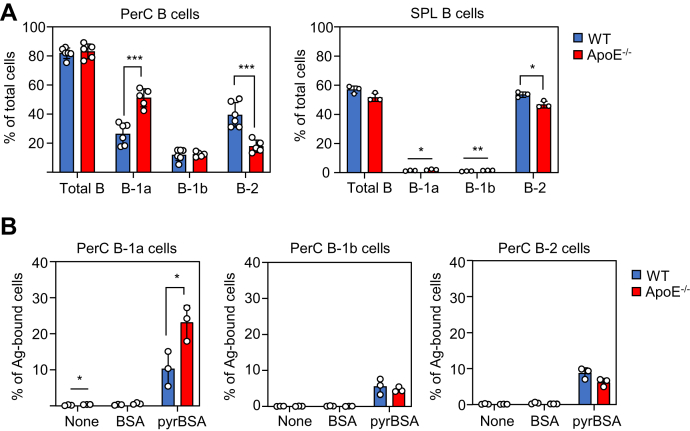
**Changes in B cell subsets and their response to pyrP in WT and apoE**^**−/−**^**mice**. *A*, the percentage of total B cells and each B cell subset (B-1a, B-1b, and B-2 cells) in the PerC (*left panel*) and SPL (*right panel*) of male WT and apoE^−/−^ mice. The cells were isolated from 21-week-old male WT and apoE^−/−^ mice and analyzed by flow cytometry. The data are shown as the mean ± SD; n = 5∼6 for PerC; n = 3 for SPL. Differences were analyzed by the unpaired Student’s *t* test; ∗*p* < 0.05; ∗∗*p* < 0.01; ∗∗∗*p* < 0.001. *B*, binding of biotinylated-BSA or biotinylated pyrBSA to the PerC B cell subsets. PerC cells isolated from the 21-week-old male WT and apoE^−/−^ mice were incubated with biotinylated BSA or biotinylated pyrBSA. The antigen-binding B-1a cells (*left panel*), B-1b cells (*middle panel*), and B-2 cells (*right panel*) are depicted as a percentage of the total PerC cells. The data are shown as the mean ± SD (n = 3). Differences were analyzed by the unpaired Student’s *t* test; ∗*p* < 0.05.

To gain insight into the enhanced innate immunity in the apoE^−/−^ mice, we analyzed changes in the B cell subsets responsible for the IgM production in the WT and apoE^−/−^ mice. The cells were isolated from both the peritoneal cavity (PerC) and spleen (SPL) of 21-week-old male WT and apoE^−/−^ mice and analyzed by flow cytometry (Figs. 3*A* and S2). The percentage of PerC B-1a cells (CD5^+^, B220^low^) was increased approximately twofold (WT: 26.4 ± 7.5%, apoE^−/−^: 51.2 ± 6.3%), whereas that of the B-2 cells (CD5^−^, B220^high^) decreased (WT: 39.4 ± 8.6%, apoE^−/−^: 17.8 ± 4.3%). Similarly, the splenic B-1a (WT: 1.4 ± 0.3%, apoE^−/−^: 2.1 ± 0.3%) and B-1b (CD5^−^, B220^low^; WT: 1.0 ± 0.2%, apoE^−/−^: 1.6 ± 0.1%) cells were upregulated but the B-2 cells decreased (WT: 53.6 ± 1.8%, apoE^−/−^: 46.8 ± 2.3%) in the apoE^−/−^ mice.

We then analyzed the changes in the proportion of the B cell subsets capable of binding pyrP. PerC cells isolated from both the male WT and apoE^−/−^ mice were treated with either biotin-labeled BSA or pyrBSA and analyzed by flow cytometry using streptavidin-APC (Fig. 3*B* and S3). No BSA-binding cells were detected in both the WT and apoE^−/−^ mice PerC B cells, whereas pyrBSA-binding cells were detected in all the B cell subsets (B-1a, B-1b, and B-2 cells). In addition, pyrBSA bound more preferentially to the B-1a cells compared with the B-1b and B-2 cells. Consistent with a significant increase in the B-1a cells in apoE^−/−^ mice compared with WT mice, the numbers of pyrBSA-binding B-1a cells were significantly higher in the apoE^−/−^ mice than in the WT mice. Binding of pyrBSA was rarely detected in the non-B cells, such as T cells (CD5^+^, B220^−^) and other cells (CD5^−^, B220^−^) (Fig. S3). Therefore, it was speculated that the B-1a cells may be involved in the promotion of the pyrrolation-specific IgM production in apoE^−/−^ mice.

### BCR-dependent IgM response to pyrP/DNA in B-1a cells

**Figure 4:**
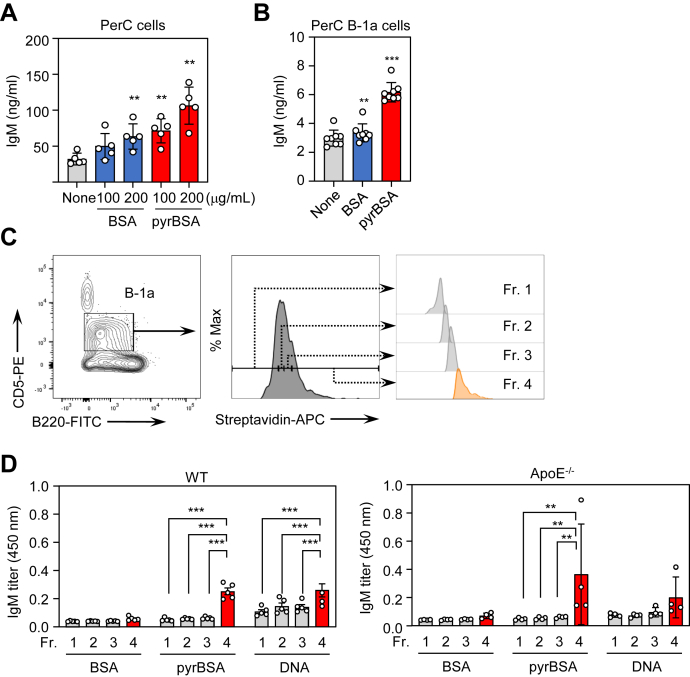
**BCR-dependent IgM response to pyrP**. *A*, effect of pyrBSA on IgM production of PerC cells *in vitro*. PerC cells were collected from 8-week-old male WT mice, seeded at 2 × 10^5^ cells/well, and stimulated with the indicated concentrations of BSA or pyrBSA. After 72 h, the total levels of IgM in the supernatants were measured by sandwich ELISA. The data are shown as the mean ± SD (n = 5). Differences were analyzed by Dunnett’s test; ∗∗*p* < 0.01; *versus* none. *B*, effect of pyrBSA on IgM production of PerC B-1a cells *in vitro*. PerC B-1a cells (CD5^+^, B220^low^) from 8-week-old male WT mice were sorted, seeded at 5 × 10^4^ cells/well, and stimulated with BSA or pyrBSA (20 μg/ml). After 84 h, the total levels of IgM in the supernatants were measured by sandwich ELISA. The data are shown as the mean ± SD (n = 8). Differences were analyzed by Dunnett’s test; ∗∗*p* < 0.01; ∗∗∗*p* < 0.001; *versus* none. *C*, representative gating strategy for identification of B-1a cells. PerC cells isolated from 21-week-old male WT and apoE^−/−^ mice were incubated with biotinylated pyrBSA. The B-1a cells (CD5^+^, B220^low^) were stained with streptavidin-APC, and subdivided into four equally sized populations (fractions 1∼4) according to the fluorescent (APC) intensity levels. *D*, the cells in each fraction isolated from the WT (*left panel*) or apoE^−/−^ mice (*right panel*) were sorted, seeded at 1 × 10^4^ cells/well, and incubated in the presence of LPS (10 μg/ml) to facilitate antibody production. After 84 h, the culture media were collected and the antibody titers against the antigens (BSA, pyrBSA, and DNA) were examined by direct antigen ELISA. The IgM concentration was quantified (data not shown) and adjusted to the same level within each assay. The data are shown as the mean ± SD (n = 5 for WT mice; n = 4 for apoE^−/−^ mice). Differences were analyzed the Tukey–Kramer test; ∗∗*p* < 0.01; ∗∗∗*p* < 0.001.

To establish that B-1a cells are involved in promoting IgM production, PerC cells were isolated from male WT mice and examined for pyrBSA-mediated IgM production *in vitro*. As shown in Figure 4*A*, treatment of PerC cells with pyrBSA showed a significant increase in the IgM production, whereas only a slight increase in the IgM production was observed with the unmodified BSA. In addition, to directly demonstrate that PerC B-1a cells can produce IgM Abs in response to pyrBSA, fluorescence-activated cell sorter (FACS)-sorted B-1a cells (CD5^+^, B220^low^) from PerC cells of WT mice were stimulated with BSA or pyrBSA, and culture supernatants were tested for the IgM production. As shown in Figure 4*B*, pyrBSA promoted the IgM production more effectively than BSA in the B-1a cells. These data indicated that the PerC B-1a cells are involved in the IgM production in response to pyrP.

We next attempted to determine the specificity of IgM produced by the pyrP-recognizing B-1a cells. To this end, B-1a cells from PerC of both the WT and apoE^−/−^ mice were treated with biotin-labeled pyrBSA, stained with streptavidin-APC, and subdivided into four equally sized populations (Fr. 1∼4), depending on the fluorescent intensity levels (Fig. 4*C*). IgM Abs produced by the B-1a cells having the highest binding capacity for pyrBSA (Fr. 4) showed significantly higher titers for pyrBSA in both the WT and apoE^−/−^ mice (Fig. 4*D*). Interestingly, IgM Abs produced by the pyrBSA-binding B-1a cells also showed a specificity toward the DNA. These data suggest the presence of pyrBSA-specific B-1a cells capable of producing bispecific IgM that cross-reacts with both pyrBSA and DNA.

### Usage of VDJ gene segments in pyrrolation-specific B-1a cells

**Figure 5:**
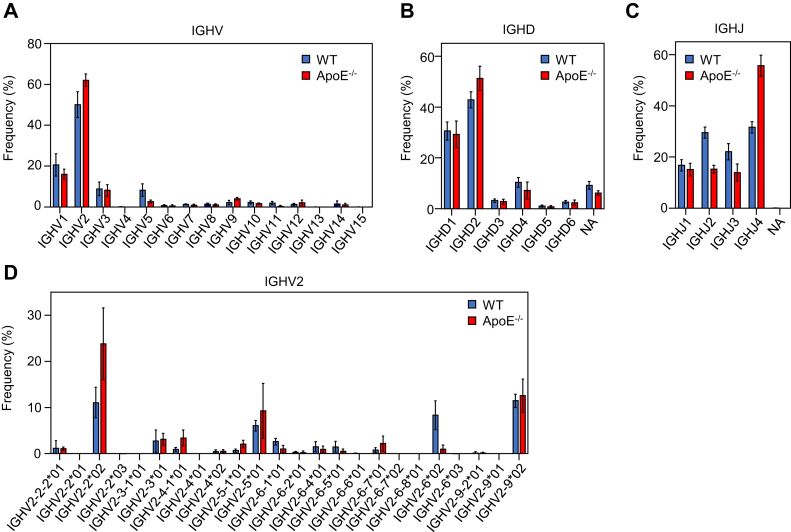
**Usage of VDJ gene segments in pyrrolation-specific B-1a cells**. *A*–*C*, PerC cells isolated from the WT mice and apoE^−/−^ mice were fractionated into the Pyr^+^ B-1a cell populations (pyrBSA-binding B-1a cells in fraction 4 of Fig. 4*C*) by flow cytometry and sequenced. Mean percentage usages of IGHV (*A*), IGHD (*B*) and IGHJ (*C*) genes are shown. Bars indicate mean percentage usage of Pyr^+^ B-1a cells of WT (*blue*) and apoE^−/−^ mice (*red*). *D*, usages of the IGHV2 gene segments. The data are shown as the mean ± SD (n = 5 for WT mice; n = 6 for apoE^−/−^ mice).

Given the presence of pyrBSA-binding B-1a cells in PerC, it was hypothesized that pyrP might associate with the membrane-bound IgM (a component of BCR) and trigger the BCR-mediated B cell activation. To gain insight into the pyrrolation-specific BCR, we sequenced the VH genes of the Pyr^+^ B-1a cells (pyrBSA-binding CD5^+^, B220^low^ cells in fraction 4 of Fig. 4*C*) isolated from PerC of male WT and apoE^−/−^ mice and compared the usage of gene segments selected from the germline gene sets, namely the immunoglobulin heavy chain variable (IGHV), immunoglobulin heavy chain diverse (IGHD), and immunoglobulin heavy chain joining (IGHJ) genes. The results showed that the Pyr^+^ B-1a cells were commonly enriched for the IGHV2 expressing clonotypes in both the WT and apoE^−/−^ mice (Fig. 5*A*). Of note, the Pyr^+^ B-1a cells of the apoE^−/−^ mice showed a more biased repertoire by using the IGHD2 gene (Fig. 5*B*) and the IGHJ4 gene (Fig. 5*C*) as compared with the WT mice.

Further analysis focusing on a set of genes that consist of the IGHV2 subgroup shows that the three genes, IGHV2-2∗02, IGHV2-5∗01, and IGHV2-9∗02, were mainly used in both the WT and apoE^−/−^ mice (Fig. 5*D*). Notably, the transcripts composed of IGHV2-2∗02 and IGHV2-5∗01were expressed at relatively high levels in the apoE^−/−^ mice, whereas the characteristic usage of IGHV2-6∗02 was observed in the WT mice (Fig. 5*D*). These data suggest that the IGHV2 gene may be critical for the Abs to bind pyrP. Given the difference in the V gene usage, there might be a difference in the structure of the IgMs expressed in the WT and apoE^−/−^ mice.

### HCDR3 characteristics of predominant BCRs expressed in pyrrolation-specific B-1a cells

**Figure 6:**
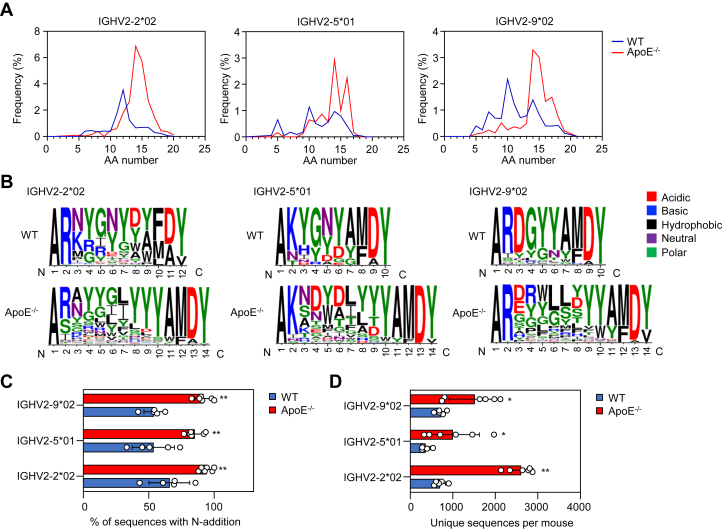
**HCDR3 characteristics of predominant BCRs expressed in pyrrolation-specific B-1a cells**. *A*, HCDR3 length distribution of VH sequences of identified IGHV families of PerC Pyr^+^ B-1a cells. The data are shown as the mean (n = 5 for WT mice; n = 6 for apoE^−/−^ mice). *B*, HCDR3 consensus logos of the mode length for pooled VH sequence using IGHV2-2∗02, IGHV 2-5∗01, and IGHV2-9∗02 segments. The size of the logo reflects the frequency of each amino acid in HCDR3. *C*, the percent of sequences with N-addition at V-D and/or D-J junctions of HCDR3 for VH sequence of the WT and apoE^−/−^ mice using IGHV2-2∗02, IGHV 2-5∗01, and IGHV2-9∗02 segments. The data are shown as the mean ± SD (n = 5 for WT mice; n = 6 for apoE^−/−^ mice). Differences were analyzed by the Mann–Whitney U test; ∗∗*p* < 0.01. *D*, the count of unique VH sequences of WT and apoE^−/−^ mice. The data are shown as the mean ± SD (n = 5 for WT mice; n = 6 for apoE^−/−^ mice). Differences were analyzed by the Mann–Whitney U test; ∗*p* < 0.05; ∗∗*p* < 0.01.

The structure of VH and light chain variable region (VL) can be subdivided into several regions. The regions responsible for the antigen contact are the heavy/light chain CDRs (HCDR1, HCDR2, and HCDR3 for the heavy chain; LCDR1, LCDR2, and LCDR3 for the light chain), flanked by framework regions (FR1, FR2, FR3, and FR4). Among them, HCDR3 is a critical region from the viewpoint of antibody specificity and diversity (16). HCDR3 is defined as the region produced by the rearrangement of the V, D, and J gene segments. During rearrangement, N-addition occurs between the V-D and D-J junctions catalyzed by the terminal deoxynucleotidyl transferase (TdT), leading to clonal diversity in the BCR repertoire (17, 18). Due to lack of the TdT expression, fetal origin B-1a cells express BCRs lacking the N-addition, whereas adult-derived B-1a cells contain an abundant N-addition (19). To compare the characteristics of the HCDR3 region of IgM-BCRs expressed by Pyr^+^B-1a cells between the male WT and apoE^−/−^ mice, we analyzed IGHV2-2∗02, IGHV2-5∗01, and IGHV2-9∗02, which were found to be the predominant genes in both the WT and apoE^−/−^ mice. As shown in Figure 6*A*, the length of HCDR3 using IGHV2-2∗02, IGHV 2-5∗01, and IGHV2-9∗02 was longer in the apoE^−/−^ mice than in the WT mice. To describe the structural properties of the BCRs, we further analyzed the sequence of HCDR3 with the most frequently occurring length (mode length) in the distribution (Fig. 6*B*). The polar amino acid pocket adjacent to the hydrophobic residues was common to the HCDR3 transcripts using IGHV2-2∗02, IGHV2-5∗01, and IGHV2-9∗02. The transcripts also contained a high proportion of polar tyrosine residues that could mediate antigen recognition upon contact with the antigens. The highly conserved arginine and lysine found in the second amino acid of HCDR3 were speculated to cause an electrostatic interaction with the negatively charged pyrP and DNA.

Furthermore, N-addition at the VDJ junctions was analyzed to determine the cause of the HCDR3 prolongation in the apoE^−/−^ mice. Interestingly, transcripts using IGHV2-2∗02, IGHV2-5∗01, and IGHV2-9∗02 showed significantly higher levels of N-addition in the apoE^−/−^ mice compared with the WT mice (Fig. 6*C*). In addition, the pyrrolation-specific BCR repertoire of the apoE^−/−^ mice was composed of more diverse sequences than the WT mice (Fig. 6*D*). Of note, the transcripts using IGHV2-6∗02 were highly conserved sequences in the WT mice, primarily without N-addition and were expressed at significantly lower levels in the apoE^−/−^ mice (Fig. S4, *A* and *C*). Like other transcripts using IGHV2 shown in Figure 6*B*, HCDR3 contained arginine and multiple tyrosine residues (Fig. S4*B*). These findings indicated that the majority of the Pyr^+^ B-1a cells in the apoE^−/−^ mice may be of adult origin.

### Bispecific IgM mAbs for pyrP/DNA

**Figure 7:**
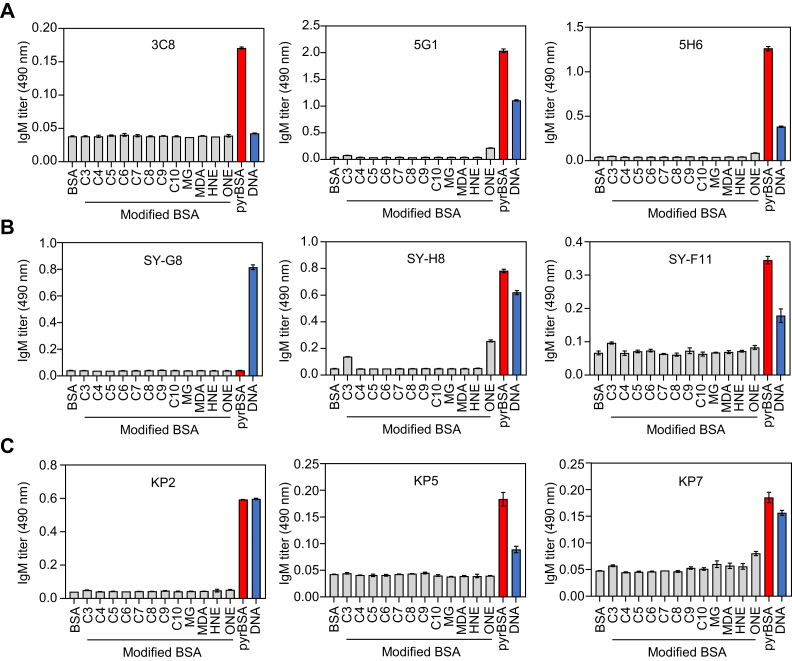
**Bispecific IgM Abs cross-reacting with both pyrP and DNA**. The antibody titers against antigens (aldehyde-treated BSAs and DNA) of IgM mAbs. The IgM mAbs produced by hybridoma clones were established from spleen cells of apoE^−/−^ mice, which are screened based on binding to pyrBSA (*A*) or DNA (*B*). The IgM mAbs produced by hybridoma clones were established from PerC Pyr^+^ B-1a cells of apoE^−/−^ mice, which are screened based on binding to pyrBSA (*C*). The IgM titers were measured by direct antigen ELISA. BSA, aldehyde-treated BSAs, and DNA were used as the coating antigens. The experiment was performed in triplicate wells. The data are shown as the mean ± SD. Aldehydes used: C3, acrolein; C4, crotonaldehyde; C5, 2-pentenal; C6, 2-hexenal; C7, 2-heptenal; C8, 2-octenal; C9, 2-nonenal; C10, 2-decenal; HNE, 4-hydroxy-2-nonenal; MDA, malondialdehyde; MG, methylglyoxal; ONE, 4-oxo-2-nonenal.

The results of the BCR repertoire analysis suggested the role for pyrP in forming the repertoire of the B-1a cell populations in the apoE^−/−^ mice. Hence, to characterize the binding specificity of IgM in detail, we isolated the hybridoma clones producing the IgM Abs specific for pyrP and/or DNA from the apoE^−/−^ mice. SPL cells from male apoE^−/−^ mice at 21 weeks of age were fused with P3/U1 murine myeloma cells, and after screening based on specific binding to pyrBSA, we established three hybridoma clones, 3C8, 5G1, and 5H6, producing IgM monoclonal Abs (mAbs) that recognize pyrBSA (Fig. 7*A*). Two of the three IgM mAbs isolated from the hybridoma clones, 5G1 and 5H6, cross-reacted with both pyrBSA and DNA, whereas 3C8 was specific for pyrP (Fig. 7*A*). The data suggest that some of the anti-pyrBSA IgM mAbs may have a common specificity toward DNA. Consistent with this hypothesis, we established three hybridoma clones from the SPL of male apoE^−/−^ mice based on the specific binding to DNA (Fig. 7*B*). Of the SY-G8, SY-H8, and SY-F11 producing the IgM mAbs specific for DNA, the two anti-DNA IgM mAbs produced from SY-H8 and SY-F11 also cross-reacted with pyrBSA (Fig. 7*B*). To further identify the pyrrolation-specific IgM produced by the PerC cells, Pyr^+^ B-1a cells isolated from the male apoE^−/−^ mice were fused with myeloma cells to establish three hybridoma clones, KP2, KP5, and KP7. IgM mAbs produced from these three hybridoma cross-reacted with both pyrBSA and DNA (Fig. 7*C*). These findings established for the first time the presence of a bispecific IgM Abs for pyrP/DNA.

### Sequence analysis of bispecific IgM mAbs for pyrP/DNA

**Figure 8:**
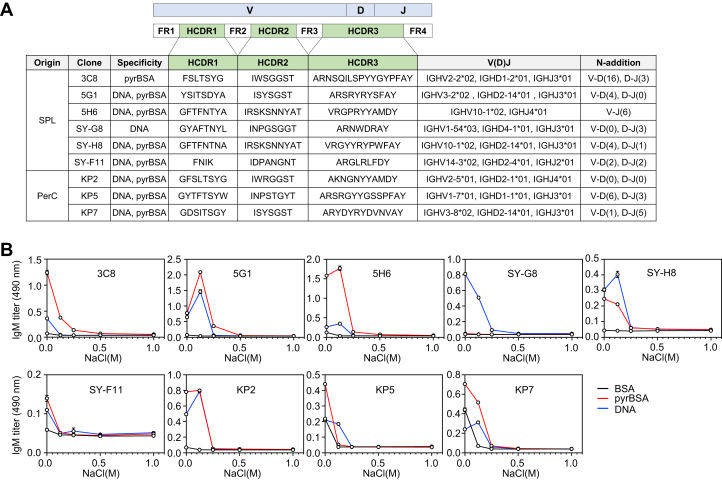
**Sequence analysis of bispecific IgM mAbs**. *A*, VDJ gene usage for VH sequence and HCDR1, HCDR2, and HCDR3 sequences from hybridomas. VH of mAbs were sequenced and the germline usages were identified using the IgBLAST and IMGT web resources. The number of N-additions at the V-D and/or D-J junctions were also identified. *B*, effect of NaCl on binding of IgM mAbs to BSA, pyrBSA, and DNA. The antigen-antibody interaction was evaluated by direct antigen ELISA. Established mAbs (3C8, 5G1, 5H6, SY-G8, SY-H8, SY-F11, KP2, KP5, and KP7) were applied to the antigen-coated 96-well plate at the indicated concentrations of NaCl. The experiment was performed in triplicate wells. The data are shown as the mean ± SD.

To characterize the genetic origin of the bispecific IgM mAbs, VH and VL genes were amplified and sequenced. As shown in Figure 8*A*, the clone KP2 expressing the bispecific IgM used the IGHV2-5∗01 gene, which was mainly used in Pyr^+^ B-1a cells in the WT and apoE^−/−^ mice (Fig. 5*D*). In addition, clone 3C8 expressing the monospecific anti-pyrBSA IgM appeared to use IGHV2-2∗02, the most predominant gene usage observed in the WT and apoE^−/−^ mice (Figs. 5*D* and 8*A*). These data suggest that the IGHV2 genes may be associated with the antigen specificity in Pyr^+^ B-1a cells. On the other hand, the presence of positively charged amino acids, such as arginine, in HCDR3 has been reported to be involved in the binding of anti-DNA autoAbs to the negatively charged phosphodiester backbone of DNA (20). Indeed, most of the IgM mAbs analyzed in this study had at least one arginine in HCDR3, but had no overlap in the V, D, and J gene selection between each clone (Fig. 8*A*). Of interest, most clones were found to contain N-additions at the V-D and D-J junctions that give rise to arginine in HCDR3 in six clones, including 5G1, 5H6, SY-G8, SY-H8, SY-F11, and KP5 (Fig. S6). This finding was consistent with the result that the pyrrolation-specific BCR repertoire of B-1a cells in the apoE^−/−^ mice was enriched in clones containing the N-addition (Fig. 6*C*). Seven clones (5G1, 5H6, SY-H8, SY-F11, KP2, KP5, and KP7) producing bispecific mAbs carried less charged amino acids, except for arginine or lysine. Pockets of arginine or lysine and polar amino acids flanked by hydrophobic residues were common to HCDR3 in all seven clones. They also contained multiple polar tyrosine residues that were found to be characteristic of HCDR3 expressed in the Pyr^+^ B-1a cells (Fig. 6*B*). Because the lysine *N*-pyrrolation confers electrical properties to proteins (14), it was speculated that the specificity of the IgM mAbs could be driven by an electrostatic interaction. This speculation was supported by the observation that the binding of the IgM mAbs to the antigens was inhibited by NaCl in a dose-dependent manner (Fig. 8*B*). Thus, the N-addition may play an important role in the antigen–antibody association by giving rise to arginine in HCDR3.

## Discussion

In our previous studies, we characterized the structural and chemical criteria governing the recognition of modified proteins by the DNA intercalators and identified γ-ketoaldehydes as the key source of the DNA mimetic proteins. Then, we established lysine *N*-pyrrolation mediated by γ-ketoaldehydes as a key covalent modification for the formation of the DNA mimetic proteins (14). 1,4-Butanedial (BDA) is the smallest γ-ketoaldehyde and exclusively forms *N*^ε^-pyrrole-L-lysine (pyrK) in proteins (14, 15). Initially, we thought that pyrK did not exist *in vivo* because BDA is a nonphysiological molecule. However, we detected pyrK *in vivo* (14, 15) and recently identified glycolaldehyde as a physiological source of pyrK (21). Therefore, pyrK was found to be a physiologically related lysine adduct. The discovery of protein *N*-pyrrolation as a novel covalent modification of lysine residues provided a rationale for establishing the molecular mechanisms and broad functional importance of the formation and regulation of pyrP. On the other hand, conversion of primary amino groups to pyrrole derivatives is also known to occur when protein lysine residues are modified with lipid peroxidation-derived aldehydes. They include 4-hydroxy-2-nonenal (22, 23), 4,5-epoxy-2-alkenals (24), and 4-oxo-2-alkenals (25). In addition, the metabolite of arachidonic acid, levuglandin E2, and unsaturated epoxyoxo fatty acids also reacts with amino groups of proteins to form pyrrole derivatives (26, 27). However, these pyrrolated derivatives derived from the lipid peroxidation-derived aldehydes are not pyrK itself, but modified lysines with alkylated pyrrole ring structures. In addition, these pyrrole-containing lysine adducts derived from the physiological aldehydes, even including glycolaldehyde-derived pyrK, are minor products, making the study of lysine pyrrolation very difficult. Therefore, the only option for studying the physiological and pathophysiological effects of lysine *N*-pyrrolation is to use BDA that can exclusively form pyrK on proteins.

The presence of an endogenous pyrrolation factor supports the idea that the processes leading to the formation of pyrP commonly take place under physiological conditions. On the other hand, it was speculated that the innate immune system may also play a role in the regulation of pyrP. In the present study, to gain insight into the physiological and pathophysiological functions of protein *N*-pyrrolation, we investigated the innate immune response in spontaneously hyperlipidemic apoE^−/−^ mice with a genetic background of BALB/c, mainly focusing on the involvement of IgM Abs in the recognition of pyrP. The resulting data showed that pyrP promotes the qualitative and quantitative changes in the BCR repertoire of B-1a cells, leading to an increased IgM response in the apoE^−/−^ mice. We also identified bispecific IgM-BCRs that respond to pyrP/DNA. The results of this study suggest the potential role of pyrP in promoting B-1a cell proliferation and the production of bispecific IgM Abs for pyrP/DNA.

An apoE deficiency has been reported to be associated with the production of autoAbs to innate antigens, such as oxidized low-density lipoproteins and numerous self-proteins (28, 29, 30). Notably, consistent with the previous studies showing the enhanced production of anti-DNA autoAbs in the apoE^−/−^ mice (15, 31), a progressive increase in the IgM response to both pyrBSA and DNA and expansion of the B-1a cells in the PerC of apoE^−/−^ mice were observed (Figs. 2 and 3*A*). Previous studies have shown that male apoE^−/−^ mice sera contain significantly higher levels of pyrK than female apoE^−/−^ mice sera (15). It is noteworthy that when the IgM titers were analyzed in both male and female mice (Fig. 2, *B* and *C*), female mice showed slightly higher levels of IgM response to pyrP than male mice. This discrepancy may be explained by the discovery that sex hormones are involved in increased production of natural antibodies in mice (32). Although the detailed mechanism of gender differences has not been investigated in this study, male mice were used for B cell analysis because male apoE^−/−^ mice are richer in pyrrolated proteins compared with female apoE^−/−^ mice. B-1 cells, a major B cell subset of PerC, spontaneously and continuously produce IgM without exogenous stimulations and maintain their numbers through self-renewal (33, 34). As B-1a cells are positively selected for their reactivity to self-antigens, the selected cells form a pool of B-1a cells to help produce the autoreactive IgM (35). The present study showed that PerC B-1a cells responded to pyrP, resulting in increased IgM production (Fig. 4, *A* and *B*). In addition, pyrBSA exhibited preferential binding to B-1a cells, and pyrBSA-binding B-1a cells, especially the Pyr^+^ B-1a cells, produced pyrrolation-specific IgMs (Figs. 3*B* and 4*D*). These findings suggest that pyrP could function as a BCR agonist stimulating IgM production by the B-1a cells.

Previous studies have shown that the PerC B-1a cells exhibit a restricted repertoire lacking diversity compared with a repertoire of other B cell subsets or B-1a cells present in other locations (36, 37). It has been speculated that self-antigens enriched in the atherosclerotic environment may affect the B cell subsets leading to changes in the BCR repertoire. However, due to the complexity of the immune response associated with the apoE deficiency, few reports have described the repertoire of B-1a cells in relation to the binding properties of IgM Abs in the apoE^−/−^ mice. In this study, we performed a BCR repertoire analysis for a qualitative description of the encoded Abs by sequencing the VH genes of the Pyr^+^B-1a cells isolated from PerC of the WT and apoE^−/−^ mice (Fig. 5). The cells showed a restricted gene usage in both the WT and apoE^−/−^ mice, especially in the IGHV2 gene segments, including IGHV2-2∗02, IGHV2-5∗01, and IGHV2-9∗02 (Fig. 5, *A* and *D*). The usage of the IGHV2-2∗02 and IGHV2-5∗01 gene segments was also observed in hybridoma clones 3C8 and KP2 (Fig. 8*A*), suggesting that the IGHV2 gene usage may be involved in the recognition of pyrP. Studies on the specificity of IgM mAbs also suggest that clones using the IGHV2-5∗01 gene segment may be associated with the bispecific IgM production in mice (Figs. 7*C* and 8*A*). Multiple factors, such as antigen availability and age, have been shown to facilitate the clonal selection of B-1a cells (38). Thus, the more biased repertoire observed in the apoE^−/−^ mice compared with the WT mice may be due in part to elevated levels of pyrP in the mice (15).

B-1a cells are derived from fetal-liver progenitors, and subsequent PerC B-1a cells express germline-encoded BCRs lacking the N-addition due to the absence of the TdT expression during B-1 cell development (18, 19). The pool of B-1a cells is maintained throughout adulthood by self-renewal (39, 40). To investigate the rearrangement associated with the apoE deficiency in the HCDR3 region of the IgM-BCRs, we compared the N-addition levels of the Pyr^+^B-1a cell in the WT and apoE^−/−^ mice (Fig. 6*C*). Our data showed that PerC B cell transcripts in the WT mice have a germline-like structure with less N-addition. Notably, transcripts with no N-addition using IGHV2-6∗02 were prominent in the WT mice but rarely expressed in the apoE^−/−^ mice (Fig. S4, *A* and *C*). In the WT mice, transcripts at the mode length of HCDR3 lack N-addition, indicating the presence of highly conserved Pyr^+^B-1a cells of fetal origin. In contrast, transcripts expressed in apoE^−/−^ Pyr^+^B-1a cells were found to use genes from the IGHV2 subgroup with significantly higher levels of N-addition in HCDR3 compared with transcripts in WT Pyr^+^B-1a cells (Fig. 6*C*), which might lead to a VH diversity (Fig. 6*D*). Repertoire discrepancies between the WT mice and apoE^−/−^ mice suggest a distinct mechanism underlying the maintenance of the B-1a populations. Indeed, the presence of clones containing the N-addition has been reported to be dependent on the specificity of BCR and exhibits different selective pressures acting on the clones, resulting in an alteration of the BCR repertoire with age (38). Previous studies have also shown that bone-marrow-derived progenitors could be a potential source of *de novo* PerC B-1a cells with N-addition (41, 42). Therefore, pyrP may help maintain the PerC B-1a populations of fetal origin by stimulating cells to a lesser extent in the WT mice compared with the apoE^−/−^ mice. The presence of highly conserved sequences may be involved in the self-replenishing of the B-1a cells in the WT mice. On the other hand, the elevated level of pyrP in the apoE^−/−^ mice may promote the *de novo* Pyr^+^ B-1a clones, leading to the expansion of clones with many N-additions.

B cells secrete IgM and exhibit the same specificity as the BCR expressed on the cell membrane. To gain insight into the pyrrolation-specific IgM-BCRs, hybridomas were generated using splenic cells and PerC cells isolated from the apoE^−/−^ mice. Two of the three hybridoma screened for specificity toward pyrP were found to produce the bispecific IgM mAb for the pyrP/DNA (Fig. 7*A*). Similarly, when establishing three hybridoma clones screened for specificity toward DNA, two of the three IgM mAbs cross-reacted with pyrP (Fig. 7*B*). These data suggest the presence of the bispecific IgM-BCRs for the pyrP/DNA. We also established hybridoma clones from the Pyr^+^ B-1a cells and found that they indeed produced the bispecific IgM (Fig. 7*C*), suggesting that the Pyr^+^ BCR repertoire shown in Figures 5 and 6 may represent the characteristics of clones that respond to both pyrP and DNA. On the other hand, to characterize the VL sequence, we sequenced the hybridomas VL and compared gene usage with the previously reported anti-DNA Abs using the IMGT web source (Fig. S7). Although the contribution of VL to the regulation of antigen binding was not well established in this study, all the identified immunoglobulin κ chain variable (IGKV) genes were identical to the IGKV genes of the anti-DNA Abs mostly established from autoimmune-prone mice (Fig. S7) (20, 43, 44, 45, 46, 47, 48). In our previous study, we showed that immunization of the BALB/c mice with pyrrolated self-molecules accelerated the production of autoAbs (14). In addition, age-dependent increases in the IgM titers as well as IgG titers for DNA and pyrP were also observed in the lupus-prone MRL-*lpr* mice (14). These data, along with current observations, suggest the association of pyrP with anti-DNA Ab response under pathological conditions.

HCDR3 is an important region for antibodies to achieve an affinity and specificity (16). To understand the characteristics of the HCDR3 of the pyrrolation-specific IgMs, we examined the structure of the HCDR3 of the Pyr^+^ B-1a cells. The results showed that the HCDR3 sequences were rich in polar amino acids flanked by the hydrophobic amino acid residues, with a positively charged, hydrophilic arginine or lysine residue, which are commonly observed in both the WT and apoE^−/−^ mice (Fig. 6*B*). Similar properties were observed in the HCDR3 of hybridoma established from the apoE^−/−^ mice (Fig. 8*A*). The fact that arginine in HCDR3 is critical for antibodies to bind to dsDNA (20) and that lysine *N*-pyrrolation alters the electronic properties of proteins (14) suggests that electrostatic interactions may contribute to the antigen–antibody interactions. Indeed, the interaction was inhibited by NaCl (Fig. 8*B*). In addition, the arginine residues of HCDR3 in several hybridoma clones were partially due to the N-addition (Fig. S6), suggesting the importance of N-addition that gives rise to arginine in the antibody recognition of antigens. The HCDR3 sequences also contained abundant tyrosine residues that were found to be higher in the apoE^−/−^ mice compared with the WT mice (Figs. 6*B* and S5). The high abundance of tyrosine in HCDR3 is an intrinsic characteristic of the Ab repertoire in murine B cells (49). Tyrosine has been reported to play an important role in recognizing antigens through hydrogen bond and hydrophobic interactions, allowing favorable contact with antigens (50, 51, 52). Thus, it can be speculated that tyrosine repeats combined with basic amino acids in HCDR3 mediate the antigen recognition of IgMs having a specificity for pyrP/DNA.

In conclusion, this study demonstrates the presence of innate B-1a cells that respond to both pyrP and DNA. The results suggest that the accumulation of pyrP due to an apoE deficiency may influence the establishment of clonal diversity in the pyrrolation-specific B cell repertoire. However, it remains unclear whether pyrP directly affects the clonal selection of immature B cells or promotes the expansion of rare clonotypes present in the peripheral mature naive B cell pool. Meanwhile, our discovery of pyrP as innate epitopes provides an important link between oxidative events and innate immunity. In addition, pyrP may serve as important signals for cell survival (also called a tonic signal) that mediate the homeostatic responses through binding to proteins (53). Future studies will be needed to investigate the mechanism of the BCR repertoire shift mediated by lysine *N*-pyrrolation to better understand the mechanism of antigen exposure and subsequent BCR selection. In addition, it is also necessary to evaluate the role of bispecific IgMs for the pyrP/DNA.

## Experimental procedures

### Materials

Bovine serum albumin (BSA) was obtained from Wako Pure Chemical Industries, Ltd (015–15103, Osaka, Japan). Calf thymus double-stranded DNA (dsDNA) was purchased from Sigma. The stock solutions of 4-hydroxy-2-nonenal (HNE) were prepared by the acid-treatment (1 mM HCl) of HNE dimethyl acetal, which was synthesized according to the procedure indicated in a previous study (54). 4-Oxo-2-nonenal (ONE) was synthesized from 2-pentylfuran according to a previous study (55). 1,4-Butanedial (BDA) was synthesized from 2,5-dimethoxytetrahydrofuran according to a previously described method (56). Malondialdehyde (MDA) was synthesized from 1,1,3,3-tetramethoxypropane according to a previous study (57). Other aldehydes were purchased from the following sources: acrolein (TCI, A0137); croton aldehyde (Wako, 039–07033); 2-pentenal (Sigma, 269255); 2-hexenal (Wako, 080-06882); 2-heptenal (Sigma, 324140); 2-octenal (Sigma, 269956); 2-nonenal (Sigma, 255653); 2-decenal (Wako, 047-20132); methylglyoxal (Sigma, M0252). Biotin-PE-maleimide was purchased from Dojindo (340-09171). All other reagents used in the study were of analytical grade and obtained from commercial sources.

### Mice

All experiments were performed according to the guidelines of the Animal Usage Committee of the Faculty of Agriculture, The University of Tokyo and were approved by the committee (Permission No. P19-047). Male, female BALB/c mice and male, female apoE-deficient mice (C.KOR/StmSlc-*Apoe*^*shl*^) were purchased from Japan SLC. The mice were housed in a temperature-controlled pathogen-free room with light from 8:00 to 20:00 (daytime) and had free access to standard food and water. Blood was collected from the tail vein and allowed to stand for 1 h at room temperature, after which the sera were collected by centrifugation at 1000*g* for 10 min and stored at –80 °C until used. All the mice were euthanized by isoflurane and sacrificed for analysis at ages between 8 and 21 weeks old.

### *In vitro* modification of BSA

Modification of the BSA by aldehydes was performed by incubating BSA (1.0 mg/ml) with aldehydes (1 mM) in PBS buffer (pH 7.4) at 37 °C. After 24 h, aliquots were collected and dialyzed against PBS. BDA was used to prepared pyrBSA.

Biotinylated BSA was prepared by adding a 10:1 M ratio of biotin-PE-maleimide to BSA. A 15-μl stock solution of biotin-PE-maleimide (50 mM stock in DMSO) was added to 1 ml of BSA (5 mg/ml) and incubated overnight at 25 °C. The aliquots were then collected and dialyzed against PBS. For preparation of the biotin-labeled pyrBSA, the biotinylated BSA was modified with 1,4-butanedial (1 mM) in PBS buffer (pH 7.4) at 37 °C under atmospheric oxygen. After 24 h, aliquots were collected and dialyzed against PBS.

### ELISA

The antibody titer of the mice sera, monoclonal antibodies, or the supernatant of cell culture was analyzed by direct antigen ELISA. BSA (50 μg/ml), aldehyde-modified BSA (50 μg/ml), and calf-thymus dsDNA (10 μg/ml) were used as the coating antigens. A 100-μl aliquot of each antigen solution was added to each well of a 96-well microtiter plate and incubated overnight at 4 °C. Subsequently, the plate was washed three times with 300 μl of PBS containing 0.5% Tween 20 (PBS/Tween) using a plate washer (Auto Mini Washer AMW-8, Biotec). Each well was incubated with 150 μl of 4% Blockace (Yukijirushi, UKB80) dissolved in distilled water for 1 h at 37 °C to block the unsaturated plastic surface. The plate was then washed in the same manner with PBS/Tween. A 100-μl aliquot of either diluted mice sera (1: 2500 dilution), diluted monoclonal antibodies (20∼50 ng/ml of IgM), or diluted supernatant of the cell culture (containing 25 ng/ml of IgM) was added to each well and incubated for 1 h at 37 °C. After discarding the supernatants and washing with PBS/Tween, 100 μl of a 1:5000 dilution of goat anti-mouse IgM conjugated to horseradish peroxidase (SouthernBiotech, 1020-05) in PBS/Tween was added. After incubation for 1 h at 37 °C, the supernatant was discarded followed by washing, and the enzyme-linked Ab bound to the well was detected by adding 100 μl/well of 1,2-phenylenediamine (0.5 mg/ml) in a 0.1 M citrate/phosphate buffer (pH 5.0) containing 0.003% hydrogen peroxide or 1-Step Ultra TMB-ELISA (Thermo Scientific, 34028). The reaction was terminated by the addition of 2 N sulfuric acid (50 μl/well), and the absorbance at 490 nm or 450 nm was read using a micro-ELISA plate reader.

The total IgM levels were measured by sandwich ELISA. The goat anti-mouse IgM (SouthernBiotech, 1020-01) was used as a capture antibody. A 100 μl of capture antibody (1:10,000 dilution) was added to each well of a 96-well microtiter plate and incubated overnight at 4 °C. The plate was then washed with PBS/Tween and blocked using 20% Blocking One (nacalai tesque, 03953-95) for 1 h at room temperature (RT). After discarding the supernatants and washing with PBS/Tween, a 100-μl aliquot of either the diluted mice sera, or diluted monoclonal antibodies, or diluted culture media was added to each well and incubated for 2 h at RT. After discarding the supernatants and washing with PBS/Tween, 100 μl of a 1:5000 dilution of goat anti-mouse IgM conjugated to horseradish peroxidase (SouthernBiotech, 1020-05) in PBS/Tween was added. After incubation for 1 h at 37 °C, the supernatant was discarded followed by washing, and the enzyme-linked Ab bound to the well was developed by 1-Step Ultra TMB-ELISA. The reaction was terminated by the addition of 2 N sulfuric acid (50 μl/well), and the absorbance at 450 nm was read using a micro-ELISA plate reader. Mouse IgM (Invitrogen, 39-50470-65) was reconstituted according to the manufacturer’s protocol. Subsequently, twofold serial dilutions of the IgM standard were performed with PBS to make the standard curve (10 ng/ml, 5 ng/ml, 2.5 ng/ml, 1.25 ng/ml, 0.625 ng/ml, 0.3125 ng/ml, 0.15625 ng/ml, and blank as 0 ng/ml).

The binding force was analyzed by ELISA in which the binding of IgM mAbs was evaluated in the presence of NaCl. Established mAbs (3C8, 5G1, 5H6, SY-G8, SY-H8, SY-F11, KP2, KP5, and KP7) were used in the assay. Briefly, a 96-well microtiter plate was coated with BSA, pyrBSA, and DNA. After blocking, each mAb was applied to the wells in the presence of the indicated concentrations of NaCl for 1 h at 37 °C. Subsequently, the bound antibody was detected as already described.

### Isolation of splenic and PerC cells

PerC cells were obtained by peritoneal lavage performed on sacrificed WT mice and apoE^−/−^ mice using 10 ml RPMI 1640+ medium (containing 10% FBS, 2 mM L-Glutamine, 100 U/ml penicillin-streptomycin). To obtain a single SPL cell suspension, mouse spleens were syringe homogenized and cells were passed through a 100-μm cell strainer using RPMI 1640+. All subsequent steps were conducted at 4 °C or on ice. The collected cells were pelleted in tubes by centrifugation at 300*g* for 5 min. The red blood cells were lysed using 1X RBC lysis buffer (Invitrogen, 00-4333-57) for 1 min. Both the PerC and SPL cells were subsequently centrifuged at 300*g* for 5 min, resuspended in the medium, and counted.

### Flow cytometry analysis and cell sorting

Cells were washed and resuspended in FACS buffer (PBS containing 2% FBS). Single cell suspensions in the FACS buffer were Fc-blocked using anti-mouse CD16/32 (TruStain fcXTM, clone: 93) at 4 °C for 15 min. Surface staining was performed using Abs including anti-mouse CD5-PE (Invitrogen, clone: 53-7.3), and anti-mouse B220-FITC (Biolegend, clone: RA3-6B2) at 4 °C for 20 min. Dead cells were identified by the scatter properties.

In some cases, biotinylated BSA or biotinylated pyrBSA was used to detect the antigen-specific B cells. Fc-blocked cell suspensions were incubated with biotinylated antigens at 4 °C for 30 min. After washing, the antigen-binding cells were detected using fluorescently labeled streptavidin (BD Pharmingen, 554067) in conjunction with the aforementioned antibodies for surface staining. Data were acquired by FACS Verse (BD Bioscience) or FACS Aria II (BD Bioscience) and subsequently analyzed using FlowJo software (BD Bioscience).

### *In vitro* cell culture

PerC cells were isolated from WT mice, seeded at 2 × 10^5^ cells/well in a 48-well plate in 500 μl of culture media (RPMI 1640+) with BSA (100 ∼ 200 μg/ml) or pyrBSA (100 ∼ 200 μg/ml) or PBS, and incubated for 72 h. The supernatant was collected for quantification of the secreted IgM.

PerC B-1a cells (CD5^+^, B220^low^) from WT mice were sorted by FACS Aria II, seeded at 5 × 10^4^ cells/well in a 48-well plate in 500 μl of culture media (RPMI 1640+) with BSA (20 μg/ml) or pyrBSA (20 μg/ml) or PBS, and incubated for 84 h. The supernatant was collected for quantification of the secreted IgM.

For a more detailed analysis of the pyrrolation-specific B-1a cells, PerC B-1a cells from the WT mice and apoE^−/−^ mice were divided into quarters (fraction 1∼4) based on their affinity to the biotinylated pyrBSA and sorted by FACS Aria II. Subsequently, the sorted cells were seeded at 1 × 10^4^ cells/well in a 48-well plate in 500 μl of culture media (RPMI 1640+) with 10 μg/ml lipopolysaccharide (LPS; Sigma, L7770) to facilitate antibody production (58). After 84 h of incubation, the supernatant was collected for the analysis of the IgM concentration and IgM titer. Briefly, the total IgM concentration of the culture media was determined by sandwich ELISA. Subsequently, the specificity toward DNA and pyrBSA of the IgMs was evaluated by direct antigen ELISA. The concentration of IgM was adjusted to the same level within each assay in order to characterize the presence of the antigen-specific B-1a cell clones.

### RNA isolation, full-length double-stranded cDNA preparation, and PCR for BCR repertoire analysis

The total B-1a cells (CD5^+^, B220^low^) and Pyr^+^ B-1a cells (CD5^+^, B220^low^ cells in fraction 4 of Fig. 4*C*) were isolated from PerC of 21-week-old male WT mice (n = 5) and apoE^−/−^ mice (n = 6). The total RNA was prepared from isolated B-1a cells using NucleoSpin RNA (Takara-bio, U0955B). Subsequently, the full-length (FL) double-stranded (ds) cDNA was prepared using 3 ng of RNA and the TeloPrime Full-Length cDNA Amplification Kit V2 (Lexogen, Inc, 013.024) according to the manufacturer’s protocol. The resulting FL ds cDNA was then globally amplified by the first PCR using reagents and 5′ and 3′ linker-specific primers included in the kit. The first PCR products were then purified with AMPure XP (Beckman Coulter, A63880). Next, the second PCR was performed using 5 ng of purified first PCR product, KOD One PCR Master Mix -Blue- (TOYOBO, KMM-201), 5′ linker-specific primer and 3′ Igμ-specific primer containing the Illumina sequencing adapters. The first and second PCR were performed using the following primers. The primers for the second PCR contain the Illumina adaptor sequences (shown in bold).

(i) first PCR: linker-specific forward primer, 5′- TGGATTGATATGTAATACGACTCACTATAG-3’; linker-specific reverse primer, 5′-TCTCAGGCGTTTTTTTTTTTTTTT TTT-3′
(ii) second PCR: forward primer, 5′-GTCTCGTGGGCTCG GAGATGTGTATAAGAGACAGTGGATTGATATGTA ATACGACTCACTATAG-3’; Igμ-chain-specific reverse primer, 5′-TCGTCGGCAGCGTCAGATGTGTATAAGA GACAGGGGGAAGACATTTGGGAAGGAC-3′

The first and second PCR were performed under the following conditions.

(i) first PCR: 1 cycle of 95.8 °C for 30 s, 50 °C for 45 s, 72 °C for 20 min; 22 cycles of 95.8 °C for 30 s, 58 °C for 30 s, 72 °C for 20 min; 72 °C for 20 min.
(ii) second PCR: 20 cycles of 95.8 °C for 30 s, 65 °C for 10 s, 68 °C for 10 s.

Subsequently, the third PCR was performed using 1 ng of purified second PCR product in order to add unique index sequences using the Nextera XT Index Kit (Illumina, FC-131-1001). Sequencing was performed at the Research Center for Environmental Quality Management of Kyoto University on the Illumina Miseq using a paired-end read sequencing with a length of 2 × 300 bp. The output sequence data were analyzed using R (version 4.1.0). The identification of the V, D, and J regions was conducted using IMGT/HighV-QUEST and IMGT web resources (59). The identical V, D, J, and DNA sequences encoding VH were defined as a unique sequence read. Before processing the data, enrichment of the sequences was evaluated by comparing the frequencies of each unique sequence between the total B-1a cells and Pyr^+^ B-1a cells (data not shown). The amino acid sequence of HCDR3 was displayed as a consensus logo using WebLogo3 (http://weblogo.threeplusone.com/) (60).

### Preparation of IgM mAbs against DNA and pyrBSA

Spleen cells from a 14-week-old male apoE^−/−^ mouse and 21-week-old male apoE^−/−^ mouse were fused with P3/U1 murine myeloma cells and cultured in hypoxanthine/aminopterin/thymidine selection medium. The Pyr^+^ B-1a cells were sorted from a pool of PerC cells of 21-week-old male apoE^−/−^ mice (n = 3) by FACS and cultured in the presence of LPS (1 μg/ml). After a 90-h incubation, the stimulated cells were fused with P3/U1 mouse myeloma cells. Hybridoma cells, corresponding to the supernatants that were positive on DNA or pyrBSA or both and negative on the native BSA, were then cloned by limiting dilution. After repeated screenings, two clones showing the most distinctive recognition of either DNA or pyrBSA and seven clones showing the most distinctive recognition of both DNA and pyrBSA were obtained. The supernatant of the cell culture was collected and mAbs were purified using a HiTrap IGM purification HP column (Cytiva, Sweden,17-5110-01) according to the manufacturer’s protocol. Subsequently, the purified mAb from each clone was dialyzed against PBS.

### Hybridoma sequence analysis

Immunoglobulin variable region genes were sequenced following amplification by PCR. The total RNA was prepared from hybridoma cells by the FastGene RNA Basic Kit (NIPPON Genetics Co, Ltd) according to the manufacturer's protocol. The cDNA synthesis was performed with ReverTra Ace qPCR RT Master Mix (TOYOBO, FSQ-301) following the manufacturer's protocol. Subsequently, variable region genes were amplified using KOD One PCR Master Mix -Blue- (TOYOBO, KMM-201), forward primers homologous to the mouse heavy and light chain leader sequences and reverse primers, as previously described (61, 62). The amplification products were subsequently Sanger sequenced. The sequence analysis tool IgBLAST and IMGT web resources were used to identify the V-(D)-J gene usage and HCDR3 junctions (59, 63).

### Statistical analysis

Statistical analyses were performed using GraphPad Prism. The data represent the mean ± the standard deviation of mean (SD) where indicated. A statistical analysis was performed by the two-tailed unpaired Student’s *t* test, one-way ANOVA or two-way ANOVA followed by Tukey–Kramer test or Dunnett’s test or Bonferroni’s test, and Mann–Whitney U test. A statistical significance indicated: ∗*p* < 0.05, ∗∗*p* < 0.01, ∗∗∗*p* < 0.001.

## Data availability

All data are included within this article.

## Supporting information

This article contains supporting information.

## Conflict of interest

The authors declare that they have no conflicts of interest with the contents of this article.
