# Supplementary material for: Unique B-1 cells specific for both N-pyrrolated proteins and DNA evolve with apolipoprotein E deficiency
Source: J Biol Chem. 2022 Jan 11;298(2):101582. doi: 10.1016/j.jbc.2022.101582 (PMC8844855; doi:10.1016/j.jbc.2022.101582)
Supplement: Supplemental Figures S1–S7 [file mmc1.pdf]

Supporting information

Unique B-1 cells specific for both *N*-pyrrolated proteins and DNA evolve with apolipoprotein E deficiency

Sei Young Lim[1], Kosuke Yamaguchi[1], Masanori Itakura[1], Miho Chikazawa[1], Tomonari Matsuda[2], and Koji Uchida[1, 3] *

[1]Graduate School of Agricultural and Life Sciences, The University of Tokyo, Tokyo 113-8657, Japan
[2]Research Center for Environmental Quality Management, Kyoto University, Otsu, Shiga, Japan
[3]Japan Agency for Medical Research and Development, CREST, Tokyo, Japan

[*]To whom correspondence should be addressed: E-mail: a-uchida@g.ecc.u-tokyo.ac.jp

Table of Contents

Figure S1. IgM response against aldehyde-modified proteins and DNA.
Figure S2. Representative flow-cytometry plots of B cell subsets in PerC and SPL.
Figure S3. Representative flow-cytometry plots of the pyrBSA-binding PerC cells.
Figure S4. HCDR3 characteristics of IgM-BCRs using the IGHV2-6*02 segment.
Figure S5. Count distribution of tyrosine residue in HCDR3 of IgM-BCRs using the IGHV2-2*02, IGHV2-5*01, and IGHV2-9*02 segments.
Figure S6. Alignments of HCDR3 junction of hybridomas with translation.
Figure S7. Sequence analysis for mAbs (VL).

Supplementary Figures

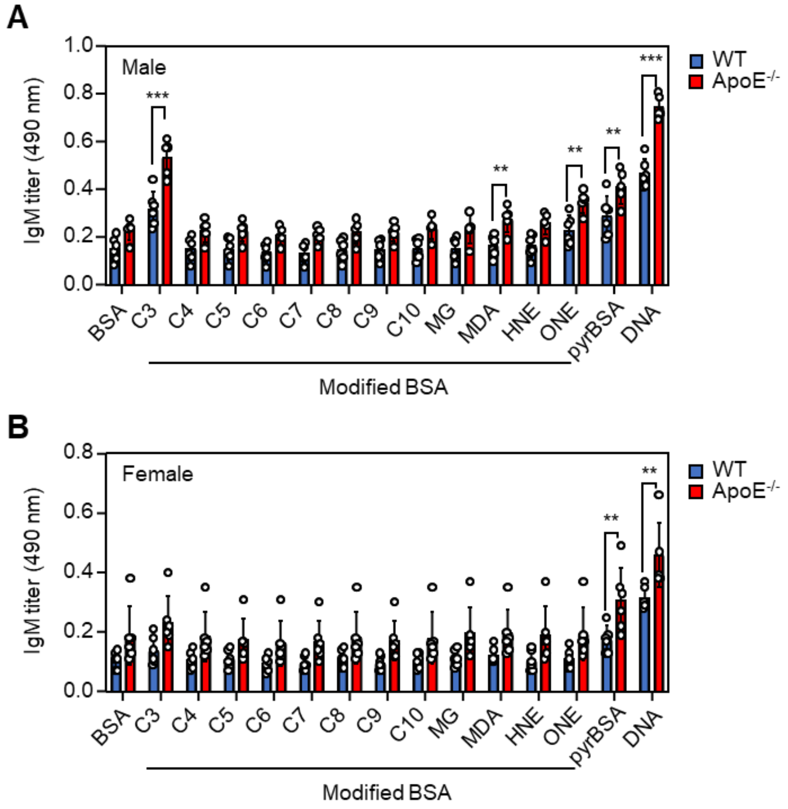

Figure S1. IgM response against aldehyde-modified proteins and DNA.
(A and B) The levels of the antibody titers against antigens (aldehyde-treated BSAs and DNA) in the mice sera. The IgM titer was measured by direct antigen ELISA with the sera of the WT mice and apoE$^{-/-}$ mice. The response in the sera of 21-week-old male mice **(A)** and 20-week-old female mice **(B)** was tested. BSA, aldehyde-treated BSA and DNA were used as the coating antigens. The data are shown as the mean ± SD (n=5~6). Differences were analyzed by two-way ANOVA adjusted by Bonferroni's multiple comparison test; ** p<0.01; *** p<0.001 for each antigen. Aldehydes used: C3, acrolein; C4, crotonaldehyde; C5, 2-pentenal; C6, 2-hexenal; C7, 2-heptenal; C8, 2-octenal; C9, 2-nonenal; C10, 2-decenal; HNE, 4-hydroxy-2-nonenal; MDA, malondialdehyde; MG, methylglyoxal; ONE, 4-oxo-2-nonenal.

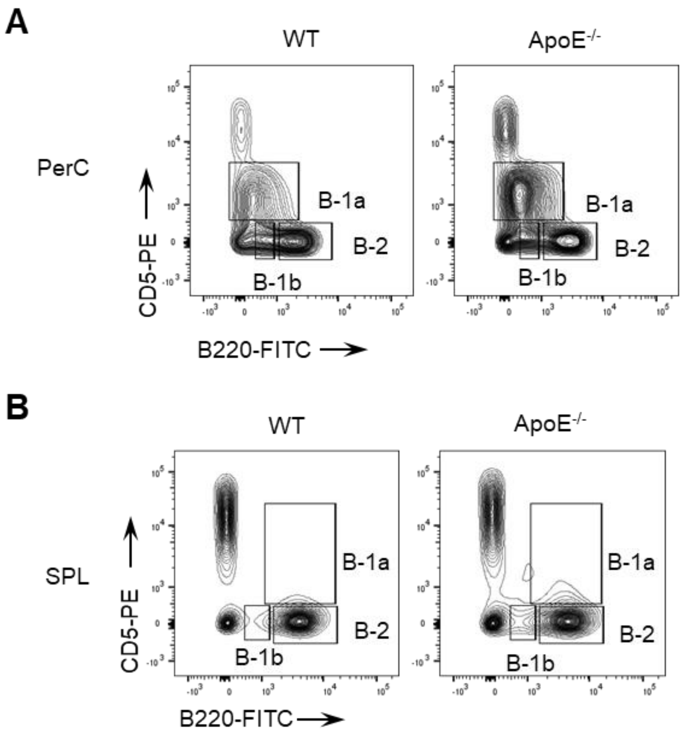

Figure S2. Representative flow-cytometry plots of B cell subsets in PerC and SPL. The gating strategy for B-1a cells ($CD5^{+}$, $B220^{low}$), B-1b cells ($CD5^{-}$, $B220^{low}$), and B-2 cells ($CD5^{-}$, $B220^{high}$) in the PerC **(A)** and SPL **(B)** isolated from the WT and apo$E^{-/-}$ mice.

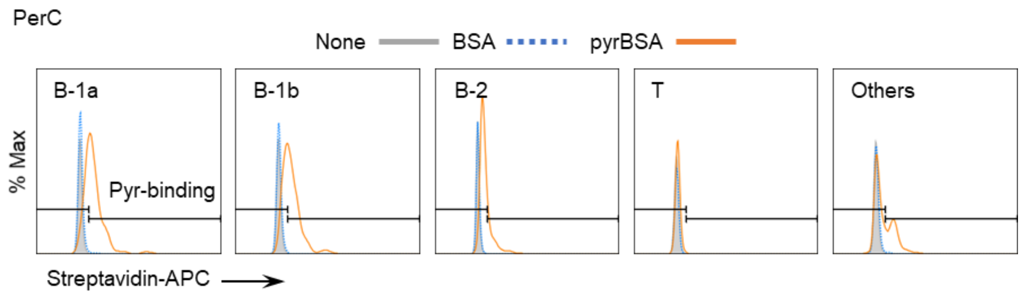

Figure S3. **Representative flow-cytometry plots of the pyrBSA-binding PerC cells.** PerC cells of the WT and apoE$^{-/-}$ mice were incubated with biotinylated BSA or biotinylated pyrBSA to evaluate the binding of antigens on cells. The PerC cells were sorted into populations of B-1a cells (CD5^{+}, B220^{low}), B-1b cells (CD5^{-}, B220^{low}), B-2 cells (CD5^{-}, B220^{high}), T cells (CD5^{+}, B220^{-}), and other cells (CD5^{-}, B220^{-}). Subsequently, the antigen-binding cells were stained using streptavidin-APC. The cells displaying a high fluorescent intensity were gated as pyrBSA-binding cells.

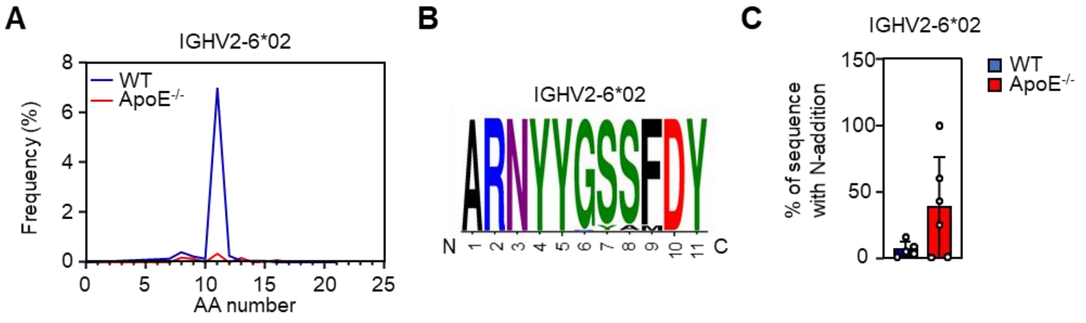

Figure S4. HCDR3 characteristics of IgM-BCRs using the IGHV2-6*02 segment. (**A**) HCDR3 length distribution of VH sequences of the identified IGHV gene of PerC Pyr^{+} B-1a cells. The data are shown as the mean (n=5 for WT mice; n=6 for apoE$^{-/-}$ mice). (**B**) HCDR3 consensus logo of the mode HCDR3 length for the pooled VH sequence using IGHV2-6*02 in the WT mice (n=5). The size of the logo reflects the frequency of each amino acid in HCDR3. (**C**) The percent of sequences with N-addition at the V-D and/or D-J junctions of HCDR3 for the VH sequence of the WT and apoE$^{-/-}$ mice. The data are shown as the mean ± SD (n=5 for WT mice; n=6 for apoE$^{-/-}$ mice).

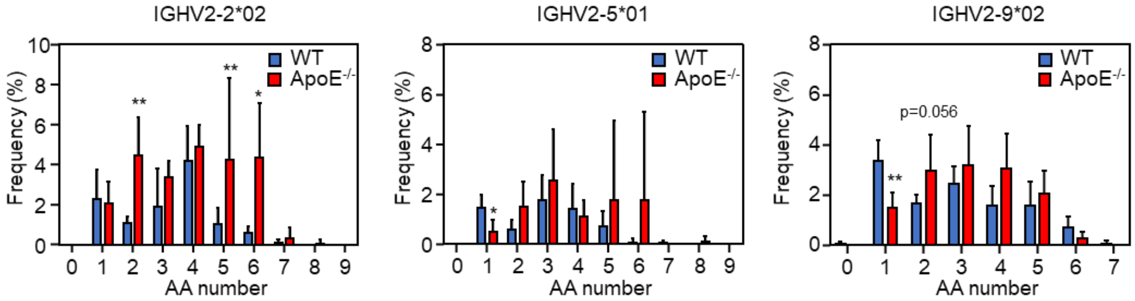

Figure S5. Count distribution of tyrosine residue in HCDR3 of IgM-BCRs using the IGHV2-2*02, IGHV2-5*01, and IGHV2-9*02 segments.
The count distribution of the tyrosine residue in HCDR3 of the VH sequences of identified IGHV families of the PerC Pyr^{+} B-1a cells. The usage of the tyrosine residue was examined in HCDR3 of IgM-BCRs using the IGHV2-2*02 (*left panel*), IGHV2-5*01(*middle panel*), and IGHV2-9*02 (*right panel*) segments. The data are shown as the mean ± SD (n=5 for WT mice; n=6 for apoE$^{-/-}$ mice). Differences were analyzed by Mann-Whitney U test; *$p < 0.05$; **$p < 0.01$.

Clone	Alignment															
3C8	A	R	N	S	Q	I	L	S	P	Y	Y	G	Y	P	F	A	Y
	gcc	aga	aat	t**cc**	**caa**	**atc**	**ctc**	**tcc**	**cc**t	tac	tac	ggc	tac	**cct**	ttt	gct	tac
5G1	A	R	S	**R**	Y	R	Y	S	F	A	Y	-	-	-	-	-	-
	gca	aga	tc**g**	**agg**	tat	agg	tac	tcg	ttt	gct	tac	-	-	-	-	-	-
5H6	V	R	G	P	**R**	Y	Y	A	M	D	Y	-	-	-	-	-	-
	gtg	aga	ggc	**ccc**	**cgg**	tac	tat	gct	atg	gac	tac	-	-	-	-	-	-
SY-G8	A	R	N	W	D	**R**	A	Y	-	-	-	-	-	-	-	-	-
	gca	aga	aac	tgg	gac	**agg**	gct	tac	-	-	-	-	-	-	-	-	-
SY-H8	V	**R**	G	Y	Y	R	Y	P	W	F	A	Y	-	-	-	-	-
	gtg	ag**g**	**ggg**	tac	tat	agg	tac	**c**cc	tgg	ttt	gct	tac	-	-	-	-	-
SY-F11	A	**R**	G	L	R	L	F	D	Y	-	-	-	-	-	-	-	-
	gct	ag**g**	**g**ga	tta	cga	c**tg**	ttt	gac	tac	-	-	-	-	-	-	-	-
KP2	A	K	N	G	N	Y	Y	A	M	D	Y	-	-	-	-	-	-
	gcc	aaa	aat	ggt	aac	tac	tat	gct	atg	gac	tac	-	-	-	-	-	-
KP5	A	R	S	**R**	G	Y	Y	G	S	S	P	F	A	Y	-	-	-
	gca	aga	tct	**cga**	**ggg**	tac	tac	ggt	agt	agc	**ccc**	ttt	gct	tac	-	-	-
KP7	A	R	Y	D	Y	R	Y	D	V	N	V	A	Y	-	-	-	-
	gca	aga	tat	**g**ac	tat	agg	tac	gac	gt**a**	**aac**	**g**tt	gct	tac	-	-	-	-

Figure S6. Alignments of HCDR3 junction of hybridomas with translation.
The nucleotide sequences and amino acid sequences in HCDR3 of the hybridomas. The N nucleotides are underlined and shown in bold. The arginine residues that originated from the N-addition are shown in bold.

A

Clone	Specificity	LCDR1	LCDR2	LCDR3	VJ
3C8	pyrBSA	QDISNY	YTS	QQGNTLPRT	IGKV10-96*01, IGKJ1*02
5G1	DNA, pyrBSA	QNVGTN	SAS	QQYNSYPLT	IGKV6-15*01 , IGKJ4*01
5H6	DNA, pyrBSA	QDIGSS	ATS	LQYASSPLT	IGKV9-120*02, IGKJ5*01
SY-G8	DNA	QSIVHSNGNTY	KVS	FQGSHVPWT	IGKV1-117*01, IGKJ1*01
SY-H8	DNA, pyrBSA	QDINKY	YTS	LQYDNLYT	IGKV19-93*01, IGKJ2*01
SY-F11	DNA, pyrBSA	QSVLYSSNQKN	WAS	HQYLSSLT	IGKV8-27*01, IGKJ5*01
KP2	DNA, pyrBSA	SSISSNY	RTS	QQGSSIPRT	IGKV4-91*01, IGKJ2*01
KP5	DNA, pyrBSA	QSLLNSSNQKNY	FAS	QQHYSTPYT	IGKV8-24*01, IGKJ2*01
KP7	DNA, pyrBSA	QSIVHSNGNTY	KVS	FQGSHVPPT	IGKV1-117*01, IGKJ1*01

B

VJ	Reference
IGKV10-96*01,IGKJ1*01	(20) Krishnan et al., 1996
IGKV6-15*01,IGKJ1*01	(43) Wloch et al., 1996
IGKV9-120*02,IGKJ2*01	(43) Wloch et al., 1996
IGKV1-117*01,IGKJ1*01	(44) Krishnan and Marion,1998
IGKV19-93*02,IGKJ2*01	(45) Jethwa et al., 2000
IGKV8-27*01,IGKJ1*01	(46) Eilat et al., 1988
IGKV4-91*01,IGKJ5*01	(47) Ibrahim et al., 1995
IGKV8-24*01,IGKJ5*01	(48) Wloch et al., 1997

Figure S7. Sequence analysis for mAbs (VL).
(**A**) VJ gene usage for the VL sequence and LCDR1, LCDR2, and LCDR3 sequences of the hybridomas. The VL of mAbs were sequenced and the germline usages were identified using the IgBLAST and IMGT web resources. (**B**) VJ gene usage for the VL sequence of the previously reported anti-DNA Abs.
